# Possible impact of COVID-19 pandemic and lockdown on suicide behavior among patients in Southeast Serbia

**DOI:** 10.1515/med-2022-0488

**Published:** 2022-06-07

**Authors:** Suzana Tosic Golubovic, Olivera Zikic, Gordana Nikolic, Jelena Kostic, Maja Simonovic, Iva Binic, Uros Gugleta

**Affiliations:** Psychiatric Clinic, University Clinic Center Nis, Nis, Serbia; University of Nis, Medical Faculty, Bul. Zorana Djindjica 88, 18000 Nis, Serbia; Center for Mental Health Protection, University Clinic Center Nis, Nis, Serbia

**Keywords:** suicidal ideation, suicide attempt, COVID-19, hospital admission

## Abstract

Individuals with serious mental illness are more affected by emotional reactions, including suicidal behavior due to COVID‐19 and psychosocial consequences of pandemic. The current cross-sectional study aimed to explore the possible association of COVID-19 and suicidal behavior (suicide ideation and attempt) before and during pandemic-associated lockdown in Serbia. We retrospectively reviewed the clinical records of 104 adult psychiatric inpatients admitted at Psychiatric Clinic, University Clinic Center Niš, Serbia, after ending lockdown and compared the obtained results with 181 adult psychiatric inpatients admitted during the same period in 2019 and 2018. Suicide ideation were more frequent in 2020 comparing with 2019 and 2018 (25 vs 12.5%, vs 9.41%; *p* < 0.05). Around 28% of patients with suicide attempts were exposed daily to the information related to COVID-19 coming from social media, while this frequency was significantly lower, only 7.55% (*p* < 0.1), among patients with no suicide ideation or attempts. Adjustment disorder was more frequent among patients with suicide attempts in comparison to the patients with suicide ideation (32 vs 11%), especially in patients without suicide ideation and attempts (32 vs 0%, *p* < 0.001). Of all studied patients with suicide attempts during 2020, 60% were not in the previous psychiatric treatment before admission.

## 1 Introduction

The first case of COVID-19 in the Republic of Serbia was confirmed on March 6, 2020. The Serbian government declared a nationwide state of emergency, in response to the growing pandemic of COVID-19 in the country, on March 15, and between March 18 and May 7, 2020, the government imposed a nationwide lockdown restricting the movement of the population, except for certified needs such as work and health circumstances [1]. Individuals with serious mental illness were adversely affected by the COVID-19 epidemic. This vulnerable population is more affected by the emotional reactions to COVID-19 prevention measures, such as outbreak isolation and quarantine, resulting in fear and anxiety development and thereby increasing diseases related to stress and at the same time causing the exacerbation of preexisting mental disorders in certain individuals [2]. As a result, apart from anxiety and depression, these individuals may experience feelings such as loneliness, denial, insomnia‚ hopelessness, which may decrease their compliance with the treatment. Anxiety, fear of death, fear of losing the loved ones, loss of social connectedness, loss of employment, and homelessness are some of the social stressors that may even trigger serious mental illnesses such as depression and/or anxiety in previously healthy persons and likewise contribute to add burden to mentally ill ones. Patients receiving outpatient treatment due to serious mental illness had difficulties maintaining their regular psychiatric visits for evaluation, treatment, prescriptions, and obtaining regular medication during the lockdown. All of the aforementioned problems may lead to the expected treatment nonadherence and worsened previous mental health symptoms. Similarly, it has been suggested that psychiatric hospitals should reduce their outpatient treatment, tighten admission criteria, and shorten the length of hospital stay worldwide and in Serbia [1]. Therefore, precautions taken as a response to the outbreak could have caused relapses and behavioral changes such as hyperactivity, agitation, and self-harm [3].

Suicide risk is impacted by many different psychiatric, psychological, social, and relational factors, and the impact of the pandemic on vulnerable individuals may have led to an increased suicide risk [4]. Therefore, these preventive measures that also increase the risk of suicide and negative emotions can lead to a decrease in the well-being of individuals with serious mental illness [5]. The impact of COVID-19 pandemic on mental health might be profound, and there are suggestions that suicide rates will increase, although this scenario is not inevitable [4]. Suicide is likely to become a more pressing concern as the pandemic spreads and has long-term effects on the general population, and vulnerable groups. Preventing suicide therefore needs urgent consideration [6].

Taking into account that COVID-19 pandemic and lockdown are associated with an increase in the risk factors for several mental health conditions and that many of these are also risk factors for suicide ideation and attempt, in the present study, we tried to evaluate whether the frequency of suicide ideation and suicide attempts differed in psychiatric hospital admissions before and during the COVID-19 pandemic and lockdown restrictions. Also, we evaluated whether patients differed in terms of sociodemographic data, diagnosis at hospital discharge, number of previous hospitalizations, reasons of admission, period of illness duration, and characteristics of the previous suicide attempts.

## 2 Methods

In this study, we performed a retrospective review of the clinical records from 104 adult psychiatric inpatients admitted at Psychiatric Clinic, University Clinic Center Niš, Serbia, from May 2020 to August 2020. This was the period after the end of the lockdown in Serbia on May 7, 2020. To compare the obtained results, we also reviewed the clinical records from 181 adult psychiatric inpatients admitted at the same clinic from May 2019 to August 2019 and the same period during 2018.

Inclusion criteria were adult inpatients aged ≥18 years hospitalized to the clinic during the mentioned period of time, and psychiatric diagnosis based on International Classification of Diseases 10th Revision (ICD X), edition 16. Exclusion criteria included the presence of dementia at admission. We prepared, following the study’s main objectives, an ad hoc checklist to gather the principal characteristic of the sample. The following variables were collected from clinical records: sociodemographic data (age, sex, marital status, having children, education, and employment), as well as clinical characteristics (diagnosis based on ICD X at hospital discharge, number of hospital admissions, period of illness evolution, and reason for the last hospital admission). Also, the presence of suicide attempt a minimum 1 year before actual hospitalization, the presence of suicide ideation, the presence of suicide attempt as a reason for the last hospital admission, and type of suicide attempt (intoxication, strangulation, jumping from height, using knife or sharp object, and other ways) were traced in the records. The diagnoses, which were present among the total sample, according to ICD X were as follows: F23-Brief psychotic disorder, F20-Schizophrenia, F25.0- Schizoaffective disorders bipolar type, F25.1-Schizoaffective disorder, depressive type, F31.1-Bipolar disorder, current episode manic, F31.3-Bipolar disorder, current episode depressed, F32-Depressive episode, F33-Major depressive disorder, recurrent, F60-Personality disorders, F43.2-Adjustment disorder, F22-Delusional disorders, F70-Intellectual disabilities, and F06-Other mental disorders due to known physiological condition.

A suicide attempt is defined as a nonfatal, self-directed, potentially injurious behavior with an implicit or explicit intent to die. The behavior may or may not result in injury, and the intensity may vary, but the decision to act out the lethal intent must be present [7].

Furthermore, according to the definition of suicide ideation adopted by Posner et al. [8], it is thoughts about wish to be dead or active thoughts of wanting to end one’s life. A trained psychiatrist assessed suicide ideation when the patient arrived at the emergency department and on admission to the psychiatric ward. Informed consent has been obtained only from individuals hospitalized at 2020. The rest of the collected data was from clinical records of admissions in 2019 and 2018. The research related to human use has been complied with all the relevant national regulations, institutional policies, and in accordance with the tenets of the Helsinki Declaration, and the study has been approved by the authors’ institutional review board or equivalent committee (num. 38788/7).

We tried to evaluate the possible impact of COVID-19-related stress, regarding psycho-socio-behavior factors such as exposure to COVID-19 information on social media, experienced fear of contamination, and adherence to protective measures (wearing a protective mask out of home) on suicidal behavior (suicide attempt or suicide ideation) among subjects admitted at our clinic during 2020. The level of exposure to information about COVID-19 was evaluated by using four options: every day, once or twice per week, once or twice per month, and without information. The fear of being contaminated was evaluated by using three options: often (once or twice per week), rarely (once or twice per month), and without fear of contamination. The adherence to protective measures (wearing a protective mask out of home) was evaluated using three options: permanently, occasionally, and never wearing a mask.


**Ethical approval:** All listed authors have agreed with the submitted manuscript and have given the corresponding author the authority to act on their behalf in all matters pertaining to publication.

## 3 Statistical analysis

The analysis of the collected data was performed in IBM SPSS Statistics for Windows, Version 20.0. Armonk, NY: IBM Corp. The Chi-square test was used to compare the differences in the relative frequencies of different categories, and *Z* test for proportions was used in pairwise comparison of frequencies. The Kruskal-Wallis test as the nonparametric test was used to compare average values of age as the only continuous variable, since the Kolmogorov Smirnov test confirmed significant deviation from a normal distribution.

## 4 Results

The total number of patients recruited and their characteristics are presented in Table 1. The first group represents patients with hospital admissions in 2020, while other two groups were patients hospitalized in 2019 and 2018. The results showed that suicide ideation was significantly increased (*p* < 0.05) in 2020, when compared to the data from 2019 and 2018 (Table 1). Even the suicide attempts, as a reason for hospital admissions, were increased during 2020, compared to the data from 2019 and 2018, and significant differences were not detected.

**Table 1:** Sociodemographic and clinical characteristics of the study groups

		Year			Total	Chi square	Sig.
		2018	2019	2020			
Group	Without suicide ideation/attempt	61 (71.76%)	69 (71.88%)	53 (50.96%)	183 (63.54%)	14.547	0.006
	Suicide ideation	8 (9.41%)	12 (12.5%)	26 (25%)	46 (17.01%)		
	Suicide attempt	16 (18.82%)	15 (15.63%)	25 (24.04%)	56 (19.44%)		
Sex	a – Female	31 (36.47%)	41 (42.71%)	46 (44.66%)	118 (41.55%)	1.366	0.505
	b – Male	54 (63.53%)	55 (57.29%)	57 (55.34%)	166 (58.45%)		
Marital status	a – Never had been married	33 (38.82%)	52 (54.17%)	57 (54.81%)	142 (49.82%)	8.404	0.210
	b – Divorced	13 (15.29%)	11 (11.46%)	15 (14.42%)	39 (13.68%)		
	c – Widow	4 (4.71%)	7 (7.29%)	5 (4.81%)	16 (5.61%)		
	d – Married	35 (41.18%)	26 (27.08%)	27 (25.96%)	88 (30.88%)		
Having children	a – Yes	48 (56.47%)	42 (43.75%)	45 (44.12%)	135 (47.7%)	3.746	0.154
	b – No	37 (43.53%)	54 (56.25%)	57 (55.88%)	148 (52.3%)		
Education	a – Primary school	14 (16.67%)	13 (13.54%)	19 (18.27%)	46 (16.2%)	8.247	0.410
	b – Secondary school	54 (64.29%)	64 (66.67%)	73 (70.19%)	191 (67.25%)		
	c – Higher school	4 (4.76%)	1 (1.04%)	3 (2.88%)	8 (2.82%)		
	d – University	12 (14.29%)	17 (17.71%)	9 (8.65%)	38 (13.38%)		
	e – Postgraduate study	0 (0%)	1 (1.04%)	0 (0%)	1 (0.35%)		
Employment	a – Occasional job	1 (1.18%)	2 (2.11%)	4 (3.85%)	7 (2.46%)	7.409	0.285
	b – Permanent job	23 (27.06%)	19 (20%)	15 (14.42%)	57 (20.07%)		
	c – Retired	11 (12.94%)	15 (15.79%)	11 (10.58%)	37 (13.03%)		
	d – Unemployed	50 (58.82%)	59 (62.11%)	74 (71.15%)	183 (64.44%)		
Number of hospital admissions	a – 1	48 (56.47%)	51 (53.13%)	58 (55.77%)	157 (55.09%)	6.863	0.738
	b – 2	20 (23.53%)	16 (16.67%)	26 (25%)	62 (21.75%)		
	c – 3	6 (7.06%)	13 (13.54%)	7 (6.73%)	26 (9.12%)		
	d – 4	7 (8.24%)	9 (9.38%)	6 (5.77%)	22 (7.72%)		
	e – 5	1 (1.18%)	2 (2.08%)	3 (2.88%)	6 (2.11%)		
	f – More than 5	3 (3.53%)	5 (5.21%)	4 (3.85%)	12 (4.21%)		
Period of illness evolution	a – Up to 6 months	23 (27.06%)	16 (16.67%)	26 (25%)	65 (22.81%)	17.707	0.125
	b – Up to 1 year	9 (10.59%)	5 (5.21%)	14 (13.46%)	28 (9.82%)		
	c – Up to 2 years	4 (4.71%)	5 (5.21%)	7 (6.73%)	16 (5.61%)		
	d – Up to 3 years	3 (3.53%)	10 (10.42%)	6 (5.77%)	19 (6.67%)		
	e – Up to 4 years	4 (4.71%)	1 (1.04%)	1 (0.96%)	6 (2.11%)		
	f – Up to 5 years	1 (1.18%)	5 (5.21%)	5 (4.81%)	11 (3.86%)		
	g – More than 5 years	41 (48.24%)	54 (56.25%)	45 (43.27%)	140 (49.12%)		
Reason for last hospital admission	a – Discontinuation of previous psychopharmacy treatment	16 (19.28%)	17 (17.71%)	22 (21.15%)	55 (19.43%)	5.296	0.506
	b – Irregular psychopharmacy treatment	7 (8.43%)	9 (9.38%)	12 (11.54%)	28 (9.89%)		
	c – No association with previous psychopharmacy treatment	32 (38.55%)	41 (42.71%)	29 (27.88%)	102 (36.04%)		
	d – No previous psychopharmacy treatment	28 (33.73%)	29 (30.21%)	41 (39.42%)	98 (34.63%)		
Diagnosis at hospital discharge	a – F23	13 (15.29%)	10 (10.42%)	13 (12.5%)	36 (12.63%)	43.489	0.009
	b – F20	17 (20%)	33 (34.38%)	37 (35.58%)	87 (30.53%)		
	c – F25.1	2 (2.35%)	1 (1.04%)	1 (0.96%)	4 (1.4%)		
	c – F25.0	9 (10.59%)	7 (7.29%)	2 (1.92%)	18 (6.32%)		
	d – F31.3	0 (0%)	4 (4.17%)	2 (1.92%)	6 (2.11%)		
	d – F31.1	4 (4.71%)	2 (2.08%)	2 (1.92%)	8 (2.81%)		
	e – F32	4 (4.71%)	3 (3.13%)	1 (0.96%)	8 (2.81%)		
	f – F33	13 (15.29%)	9 (9.38%)	6 (5.77%)	28 (9.82%)		
	g – F60	8 (9.41%)	7 (7.29%)	13 (12.5%)	28 (9.82%)		
	h – F43.2	2 (2.35%)	5 (5.21%)	11 (10.58%)	18 (6.32%)		
	i – F22.0	2 (2.35%)	5 (5.21%)	8 (7.69%)	15 (5.26%)		
	j – F70	0 (0%)	4 (4.17%)	0 (0%)	4 (1.4%)		
	k – F06	11 (12.9%)	6 (6.25%)	8 (7.69%)	25 (8.8%)		
Age		45.34 ± 14.06	40.70 ± 14.73	36.67 ± 13.88	40.58 ± 13.88	18.485	0.000

Undernote: ICD X diagnosis codes: F23 – Brief psychotic disorder, F20 – Schizophrenia, F25.0 – Schizoaffective disorders bipolar type, F25.1 – Schizoaffective disorder, depressive type, F31.1 – Bipolar disorder, current episode manic, F31.3 – Bipolar disorder, current episode depressed, F32 – Depressive episode, F33 – Major depressive disorder, recurrent, F60 – Personality disorders, F43.2 – Adjustment disorder, F22 – Delusional disorders, F70 – Intellectual disabilities, F06 – Other mental disorders due to known physiological condition.

As shown in Table 1, the obtained results indicate a significantly lower percentage of admitted patients without suicide ideation and suicide attempt in 2020, compared to the data from 2019 and 2018 (*p* < 0.01). Conversely, the most frequent diagnosis at discharge, among the total sample of patients, was schizophrenia (F20-ICD X). Also, this diagnosis was markedly frequent in patients admitted in 2020 compared to those admitted in 2018 (*p* < 0.05), and when compared with 2019 admissions, the frequency was similar and not statistically different. Major depressive disorder, recurrent (F33 ICD X), was significantly lower (*p* < 0.05) in patients from 2020 compared to the patients from 2018, while no significant difference was obtained when compared with patients from 2019.

Schizoaffective disorders, bipolar type (F25.0-ICD X), and average age of the patients in 2020 showed significant lower levels (*p* < 0.05; *p* < 0.001, respectively) compared with patients from 2019 and 2018. Furthermore, adjustment disorder (F43.2-ICD X) was significantly higher (*p* < 0.05) among patients in 2020, compared to the patients from 2019 and 2018. The diagnoses of PTSD, as well as addiction disorders, were not present in the total sample. The rest of sociodemographic and clinical characteristics, presented in Table 1, showed no significant differences between the evaluated groups.

Based on the results obtained in our study, patients hospitalized after lockdown in 2020 were divided into three groups, including patients without suicide ideation and suicide attempt, patients with suicide ideation, and patients with suicide attempts. Evaluation of the possible impact of COVID-19-related stress showed that patients with suicide attempts were significantly (*p* < 0.1) exposed each day to information about COVID-19 in social media, compared to the patients without suicide ideation and attempt (Table 2).

**Table 2:** Sociodemographic and clinical characteristics of subjects admitted after lockdown in Serbia

		Reason for hospital admission			Total	Chi square	Sig.
		Without suicide ideation and attempt	With suicide ideation	With suicide attempt			
Exposure to COVID-19 information	a – Each day	4 (7.55%)	4 (15.38%)	7 (28%)	15 (14.42%)	10.775	0.096
	b – Occasional – once to twice per week	14 (26.42%)	10 (38.46%)	9 (36%)	33 (31.73%)		
	c – Rear – once to twice per month	18 (33.96%)	6 (23.08%)	7 (28%)	31 (29.81%)		
	d – No information	17 (32.08%)	6 (23.08%)	2 (8%)	25 (24.04%)		
Experienced fear of contamination	a – Often – 1–2 days per week	3 (5.66%)	5 (19.23%)	9 (36%)	17 (16.35%)	25.019	0.000
	b – Rear – 1–2 days per month	13 (24.53%)	9 (34.62%)	13 (52%)	35 (33.65%)		
	c – No fear	37 (69.81%)	12 (46.15%)	3 (12%)	52 (50%)		
Adherence to protection measures	a – Fully complied	4 (7.55%)	8 (30.77%)	4 (16%)	16 (15.38%)	8.143	0.086
	b – Occasional	39 (73.58%)	16 (61.54%)	18 (72%)	73 (70.19%)		
	c – Never	10 (18.87%)	2 (7.69%)	3 (12%)	15 (14.42%)		
Sex	a – Female	24 (46.15%)	13 (50%)	9 (36%)	46 (44.66%)	1.106	0.575
	b – Male	28 (53.85%)	13 (50%)	16 (64%)	57 (55.34%)		
Marital status	a – Never had been married	37 (69.81%)	10 (38.46%)	10 (40%)	57 (54.81%)	18.527	0.005
	b – Divorced	5 (9.43%)	8 (30.77%)	2 (8%)	15 (14.42%)		
	c – Widow	3 (5.66%)	0 (0%)	2 (8%)	5 (4.81%)		
	d – Married	8 (15.09%)	8 (30.77%)	11 (44%)	27 (25.96%)		
Having children	a – Yes	14 (26.92%)	17 (65.38%)	14 (58.33%)	45 (44.12%)	12.973	0.002
	b – No	38 (73.08%)	9 (34.62%)	10 (41.67%)	57 (55.88%)		
Education	a – Primary school	10 (18.87%)	4 (15.38%)	5 (20%)	19 (18.27%)	1.905	0.928
	b – Secondary school	37 (69.81%)	20 (76.92%)	16 (64%)	73 (70.19%)		
	c – Higher school	2 (3.77%)	0 (0%)	1 (4%)	3 (2.88%)		
	d – University	4 (7.55%)	2 (7.69%)	3 (12%)	9 (8.65%)		
Employment	a – Occasional job	1 (1.89%)	1 (3.85%)	2 (8%)	4 (3.85%)	13.118	0.041, *p* < 0.05
	b – Permanent job	5 (9.43%)	2 (7.69%)	8 (32%)	15 (14.42%)		
	c – Retired	5 (9.43%)	2 (7.69%)	4 (16%)	11 (10.58%)		
	d – Unemployed	42 (79.25%)	21 (80.77%)	11 (44%)	74 (71.15%)		
Number of hospital admissions	a – 1	29 (54.72%)	13 (50%)	16 (64%)	58 (55.77%)	5.857	0.827
	b – 2	13 (24.53%)	6 (23.08%)	7 (28%)	26 (25%)		
	c – 3	4 (7.55%)	2 (7.69%)	1 (4%)	7 (6.73%)		
	d – 4	4 (7.55%)	2 (7.69%)	0 (0%)	6 (5.77%)		
	e – 5	1 (1.89%)	2 (7.69%)	0 (0%)	3 (2.88%)		
	f – More than 5	2 (3.77%)	1 (3.85%)	1 (4%)	4 (3.85%)		
Period of illness evolution	a – Up to 6 months	15 (28.3%)	1 (3.85%)	10 (40%)	26 (25%)	17.591	0.129
	b – Up to 1 year	4 (7.55%)	6 (23.08%)	4 (16%)	14 (13.46%)		
	c – Up to 2 years	2 (3.77%)	4 (15.38%)	1 (4%)	7 (6.73%)		
	d – Up to 3 years	3 (5.66%)	1 (3.85%)	2 (8%)	6 (5.77%)		
	e – Up to 4 years	1 (1.89%)	0 (0%)	0 (0%)	1 (0.96%)		
	f – Up to 5 years	3 (5.66%)	1 (3.85%)	1 (4%)	5 (4.81%)		
	g – More than 5 years	25 (47.17%)	13 (50%)	7 (28%)	45 (43.27%)		
Reason for last hospital admission	a – Discontinuation of previous psychopharmacy Treatment	13 (24.53%)	6 (23.08%)	3 (12%)	22 (21.15%)	12.721	0.048
	b – Irregular psychopharmacy treatment	8 (15.09%)	3 (11.54%)	1 (4%)	12 (11.54%)		
	c – No association with previous psychopharmacy treatment	11 (20.75%)	12 (46.15%)	6 (24%)	29 (27.88%)		
	d – No previous psychopharmacy treatment	21 (39.62%)	5 (19.23%)	15 (60%)	41 (39.42%)		
Diagnose at hospital discharge	a – F23	11 (20.75%)	0 (0%)	2 (8%)	13 (12.5%)	63.739	0.000
	b – F20	23 (43.4%)	7 (26.92%)	7 (28%)	37 (35.58%)		
	c – F25.1	0 (0%)	1 (3.85%)	0 (0%)	1 (0.96%)		
	c – F25.0	2 (3.77%)	0 (0%)	0 (0%)	2 (1.92%)		
	d – F31.3	1 (1.89%)	1 (3.85%)	0 (0%)	2 (1.92%)		
	d – F31.1	2 (3.77%)	0 (0%)	0 (0%)	2 (1.92%)		
	e – F32	0 (0%)	0 (0%)	1 (4%)	1 (0.96%)		
	f – F33	0 (0%)	4 (15.38%)	2 (8%)	6 (5.77%)		
	g – F60	4 (7.55%)	8 (30.77%)	1 (4%)	13 (12.5%)		
	h – F43.2	0 (0%)	3 (11.54%)	8 (32%)	11 (10.58%)		
	i – F22.0	8 (15.09%)	0 (0%)	0 (0%)	8 (7.69%)		
	j – F70	2 (3.77%)	2 (7.69%)	4 (16%)	8 (7.69%)		
Age		34.51 ± 12.47	37.12 ± 14.20	40.80 ± 15.87	36.67 ± 13.66	2.478	0.290

Undernote: ICD X diagnosis codes: F23 – Brief psychotic disorder, F20 – Schizophrenia, F25.0 – Schizoaffective disorders bipolar type, F25.1 – Schizoaffective disorder, depressive type, F31.1 – Bipolar disorder, current episode manic, F31.3 – Bipolar disorder, current episode depressed, F32 – Depressive episode, F33 – Major depressive disorder, recurrent, F60 – Personality disorders, F43.2 – Adjustment disorder, F22 – Delusional disorders, F70 – Intellectual disabilities, F06 – Other mental disorders due to known physiological condition.

Often the fear of contamination (1–2 times weekly) was found to be significantly (*p* < 0.001) increased in patients with suicide attempts, compared to the patients without suicide ideation and attempt. Furthermore, patients with suicide ideation permanently wear a protective mask outside of their homes, significantly more often (*p* < 0.1) compared to the patients without suicide ideation and attempt (Table 2). The results in Table 2 indicate that patients with suicide ideation show significantly lower (*p* < 0.05) psychiatric treatment before admission, including psychopharmaceutical treatment, compared to the patients with suicide attempts. Also, significantly lower number of married patients (*p* < 0.01) and patients with children (*p* < 0.01) were detected in the group of patients with suicide attempts during 2020, compared to the patients without suicide ideation and attempt. Conversely, the number of unemployed persons was significantly low (*p* < 0.05) in patients with suicide attempts compared to the patients with suicide ideation. Patients with suicide attempts diagnosed with adjustment disorder (F43.2-ICD X) were significantly higher (*p* < 0.001) when compared to patients without suicide ideation and attempt. Brief psychotic disorder (F23-ICD X) and delusional disorders (F22-ICD X) were significantly less frequent (*p* < 0.001) in patients with suicide ideation and the patients with suicide attempts compared to the patients without suicide ideation and attempt.

Major depressive disorder, recurrent (F33-ICD X), in patients with suicide ideation and patients with suicide attempts was significantly higher (*p* < 0.001) compared with patients without suicide ideation and attempt. Personality disorders (F60-ICD X) in patients with suicide ideation were significantly higher (*p* < 0.001), compared with patients without suicide ideation and attempt. The rest of sociodemographic and clinical characteristics, presented in Table 2, showed no significant differences between the evaluated groups.

Finally, the data comparison of all patients with suicide attempts regarding the year of admission was performed. The first group includes the patients hospitalized after lockdown in 2020, the second group in 2019, and the third group during 2018 (Table 3).

**Table 3:** Sociodemographic and clinical characteristics of all study subjects with suicide attempt

		Years			Total	Chi square	Sig.
		2018	2019	2020			
Sex	a – Female	8 (50%)	6 (40%)	9 (36%)	23 (41.07%)	0.800	0.670
	b – Male	8 (50%)	9 (60%)	16 (64%)	33 (58.93%)		
Marital status	a – Never had been married	6 (37.5%)	6 (40%)	10 (40%)	22 (39.29%)	7.320	0.292
	b – Divorced	1 (6.25%)	1 (6.67%)	2 (8%)	4 (7.14%)		
	c – Widow	1 (6.25%)	5 (33.33%)	2 (8%)	8 (14.29%)		
	d – Married	8 (50%)	3 (20%)	11 (44%)	22 (39.29%)		
Having children	a – Yes	10 (62.5%)	10 (66.67%)	14 (58.33%)	34 (61.82%)	0.276	0.871
	b – No	6 (37.5%)	5 (33.33%)	10 (41.67%)	21 (38.18%)		
Education	a – Primary school	2 (12.5%)	1 (6.67%)	5 (20%)	8 (14.29%)	4.022	0.674
	b – Secondary school	8 (50%)	11 (73.33%)	16 (64%)	35 (62.5%)		
	c – Higher school	2 (12.5%)	1 (6.67%)	1 (4%)	4 (7.14%)		
	d – University	4 (25%)	2 (13.33%)	3 (12%)	9 (16.07%)		
Employment	a – Occasional job	0 (0%)	1 (6.67%)	2 (8%)	3 (5.36%)	10.339	0.111
	b – Permanent job	1 (6.25%)	4 (26.67%)	8 (32%)	13 (23.21%)		
	c – Retired	1 (6.25%)	4a (26.67%)	4 (16%)	9 (16.07%)		
	d – Unemployed	14 (87.5%)	6 (40%)	11 (44%)	31 (55.36%)		
Number of hospital admissions	a – 1	11 (68.75%)	9 (60%)	16 (64%)	36 (64.29%)	8.101	0.424
	b – 2	4 (25%)	2 (13.33%)	7 (28%)	13 (23.21%)		
	c – 3	0 (0%)	2 (13.33%)	1 (4%)	3 (5.36%)		
	d – 4	1 (6.25%)	2 (13.33%)	0 (0%)	3 (5.36%)		
	e – 5	0 (0%)	0 (0%)	1 (4%)	1 (1.79%)		
Period of illness duration	a – Up to 6 months	6 (37.5%)	3 (20%)	10 (40%)	19 (33.93%)	11.746	0.466
	b – Up to 1 year	3 (18.75%)	1 (6.67%)	4 (16%)	8 (14.29%)		
	c – Up to 2 years	1 (6.25%)	1 (6.67%)	1 (4%)	3 (5.36%)		
	d – Up to 3 years	0 (0%)	0 (0%)	2 (8%)	2 (3.57%)		
	e – Up to 4 years	1 (6.25%)	0 (0%)	0 (0%)	1 (1.79%)		
	f – Up to 5 years	0 (0%)	2 (13.33%)	1 (4%)	3 (5.36%)		
	g – More than 5 years	5 (31.25%)	8 (53.33%)	7 (28%)	20 (35.71%)		
Reason for last hospital admission	a – Discontinuation of previous psychopharmacy treatment	3 (18.75%)	2 (13.33%)	3 (12%)	8 (14.29%)	5.350	0.500
	b – Irregular psychopharmacy treatment	1 (6.25%)	1 (6.67%)	1 (4%)	3 (5.36%)		
	c – No association with previous psychopharmacy treatment	6 (37.5%)	8 (53.33%)	6 (24%)	20 (35.71%)		
	d – No previous psychopharmacy treatment	6 (37.5%)	4 (26.67%)	15 (60%)	25 (44.64%)		
Diagnosis at hospital discharge	a – F23	1 (6.25%)	0 (0%)	2 (8%)	3 (5.36%)	24.497	0.139
	b – F20	3 (18.75%)	3 (20%)	7 (28%)	13 (23.21%)		
	c – F25.1	2 (12.5%)	0 (0%)	0 (0%)	2 (3.57%)		
	c – F25.0	0 (0%)	2 (13.33%)	0 (0%)	2 (3.57%)		
	d – F31.3	3 (18.75%)	2 (13.33%)	1 (4%)	6 (10.71%)		
	d – F31.1	2 (12.5%)	3 (20%)	2 (8%)	7 (12.5%)		
	e – F32	2 (12.5%)	1 (6.67%)	1 (4%)	4 (7.14%)		
	f – F33	0 (0%)	3 (20%)	8 (32%)	11 (19.64%)		
	g – F60	1 (6.25%)	0 (0%)	0 (0%)	1 (1.79%)		
	h – F43.2	2 (12.5%)	1 (6.67%)	4 (16%)	7 (12.5%)		
Age		44.12 ± 10.62	44.27 ± 16.14	40.80 ± 15.87	42.68 ± 14.49	1.220	0.543
Way of suicide attempt	a – Drug intoxication	11 (68.75%)	6 (42.86%)	10 (40%)	27 (49.09%)	7.042	0.532
	b – Hanging	1 (6.25%)	2 (14.29%)	4 (16%)	7 (12.73%)		
	c – Jumping	1 (6.25%)	0 (0%)	2 (8%)	3 (5.45%)		
	d – Using knife or sharp object	1 (6.25%)	5 (35.71%)	7 (28%)	13 (23.64%)		
	e – Other	2 (12.5%)	1 (7.14%)	2 (8%)	5 (9.09%)		

Undernote: ICD X diagnosis codes: F23 – Brief psychotic disorder, F20 – Schizophrenia, F25.0 – Schizoaffective disorders bipolar type, F25.1 – Schizoaffective disorder, depressive type, F31.1 – Bipolar disorder, current episode manic, F31.3 – Bipolar disorder, current episode depressed, F32 – Depressive episode, F33 – Major depressive disorder, recurrent, F60 – Personality disorders, F43.2 – Adjustment disorder, F22 – Delusional disorders, F70 – Intellectual disabilities, F06 – Other mental disorders due to known physiological condition.

From the total number of patients, 60% who attempted suicide during 2020 never received psychiatric treatment before hospitalization (including psychopharmacy treatment), while this percentage was significantly lower (*p* < 0.01) during 2019. The frequency of brief psychotic disorder (F23-ICD X) in 2020 was significantly higher (*p* < 0.001) in patients without suicide ideation and attempt compared to the same diagnoses in patients with suicide attempts and suicide ideation. However, adjustment disorder (F43.2-ICD X) was significantly higher (*p* < 0.001) in patients with suicide attempts compared to the patients without suicide ideation and patients with suicide attempts (Table 3).

## 5 Discussion

The ongoing COVID-19 pandemic affects most aspects of society and represents a major psychosocial stressor whose impact on the incidence of mental disorders has already been noted [9,10,11,12]. A recent systematic review has underlined the negative psychological effects of lockdown, including various factors associated with increased suicide risk [13]. Furthermore, several reports have noted that the COVID-19 pandemic and its mitigation policies have led to factors known to precipitate suicide, including social isolation, financial stressors, intensive exposure to stories of hopelessness (through the media), emerging or exacerbated psychological and psychiatric suffering, and other factors [14,15,16,17].

The current cross-sectional study compared patients hospitalized at Psychiatry Clinic Nis, Serbia, 4 months after lockdown in 2020, regarding the frequency of suicide ideation and suicide attempts at psychiatric hospital admission, before and during the lockdown, due to COVID-19 pandemic. In contrast with other research studies [18,19,20,21], which reported a significant drop in psychiatric admissions during the COVID-19 lockdown (i.e., March 1–April 30, 2020), our results reported more hospitalizations during 4 months in 2020, comparing with the same period of time in 2019 and 2018; however, the results were not found to be statistically significant. Gómez-Ramiro et al. [22] showed a significant decrease in the number of psychiatric emergency admissions during the first 3 months of lockdown, compared to the 3 months earlier. Different observation periods and the number of days may explain the differences in the results of various research studies. Moreover, differences with other studies possibly reflect the different organization of the psychiatric units in other countries. It should be noted that we enrolled patients in the period immediately after the end of the national lockdown in Serbia because some urgent hospitalizations were delayed during the lockdown period, per the guideline given by the government.

Despite the early detection of psychological distress in the general population due to the COVID-19 pandemic, its impact on suicidal behavior and mainly on suicidal ideation has not been largely assessed in cross-sectional studies. Our study results indicated that patients with suicidal ideation were significantly more frequent in 2020 compared to the patients in 2019 and 2018. Boldrini et al. [18] reported a 35% increase in suicide ideation in patients during the postlockdown period (i.e., May 1–June 30, 2020), compared to the rates observed in 2018 and 2019. However, that study did not investigate possible differences in suicide attempts.

Suicidal ideation in the general population is a strong predictor of subsequent suicide attempts, and thus, studies have highlighted the importance of its detection in the general population to inform efforts to improve prevention strategies that would adequately address suicide ideation risk and protective factors [23]. Recent meta-analysis across 54 studies suggested increased event rates for suicide ideation, suicide attempts, and self-harm during the COVID-19 pandemic when considered against event rates from prepandemic studies [24]. However, these results are not in agreement with a recent systematic review that did not report an increase in suicide behaviors, suicide attempts, and suicide ideation during the COVID-19 pandemic [25]. Berardelli et al. [26] indicated that only suicide attempts, and not suicide ideation, were more frequent in the psychiatric hospital admission during the COVID-19 pandemic than before. The results of research on the suicide rates during the COVID-19 pandemic are presently inconsistent and reveal that the impact of COVID-19 on suicide rates has not been uniform across countries [27,28,29]. Only a few studies have investigated how epidemics affect suicidality [14,15,30].

Indisputably, the COVID-19 pandemic has influenced suffering among individuals in general [4], and several factors may have influenced suicide attempts in psychiatric patients, including the loss of employment and other factors [16]. Our results are not in line with these studies because the number of unemployed subjects with suicide attempt during 2020 was significantly lower, compared to the almost similar number of subjects with suicide ideation and subjects without suicide ideation and attempts. These findings could be possibly explained by higher distress among employed subjects due to economic repercussion of COVID-19 worldwide and possible job loss.

The average age among patients admitted in 2020 was lower compared to those admitted in 2019 and 2018, suggesting that the younger population was under great distress due to COVID-19 lockdown, quarantine, social distancing, possibility of COVID-19 contamination, and pandemic repercussions on financial and job security. Many young people have had their education interrupted and are anxious about their prospects. Earlier meta-analysis suggested that being younger and female are vulnerability factors for suicide ideation during the COVID-19 pandemic [24].

Preliminary data have suggested that psychiatric patients might experience depression, anxiety, insomnia, and other psychiatric symptoms during the COVID-19 pandemic [10,22,31], while Wang et al. [32] did not find the differences in the mentioned symptoms. In our study, the most frequent psychiatric diagnosis at hospital discharge among the total sample of patients was schizophrenia. This diagnosis was more frequent among patients hospitalized during 2020, compared to patients hospitalized in 2018, while compared to patients hospitalized in 2019, the frequency was near similar, and these findings could be explained with the fact that our institution predominantly treats schizophrenic patients. The diagnosis of adjustment disorder was significantly more frequent among patients hospitalized during 2020, compared to patients in 2019 and 2018, which indicates the importance of psychosocial implications of COVID-19 on subjects, who have never had psychiatry illness and treatment before hospital admission.

Montalbani et al. [33] reported that patients with mental disorders consulted psychiatric services less frequently during the pandemic than before, probably aggravating their emotional and psychological pain. All these factors may contribute to the increased frequency of suicide attempts reported in the present study. Several studies have reported that fear of COVID-19, uncertainty about the future, stigma, and social isolation are significant psychological stressors that may interact to determine psychopathological outcomes, including suicidality [2,13]. Prolonged social isolation during the stay-at-home directives has been associated with the increased loneliness and reduced interpersonal connectedness and social identity, potentially increasing the risk for suicide.

We tried to compare all admissions during the mentioned period in 2020, according to the presence of suicide ideation, attempts, and all other reasons for admission, as well as on questions on sociodemographic, clinical, and variables due to COVID-19, which was not evaluated, according to our knowledge, in previous studies. More than a quarter of patients with suicide attempt each day was exposed to the information in social media about COVID-19, while this frequency was significantly lower among patients with no suicide ideation or attempt. Repeated exposure to the information about the COVID-19 crisis can intensify anxiety, fear, depression, and sleep disorders and heighten the suicide risk [14]. Anxiety and fear of contagion during the COVID-19 crisis may be related to uncertainty, fear of unknown, and panic-inducing stories in traditional and social media [34]. Stressful factors were identified, including quarantine length >10 days, fear of contracting the infection, boredom and frustration for forced inactivity, and fear of shortness of essential elements of survival especially in the case of further prolongation of the institution [13].

Often the fear of being contaminated (1–2 times weekly) reported about one-third of patients with suicide attempts and one-fifth of patients with suicide ideation, while this frequency was significantly lower among patients without suicide ideation or attempt. About a third of patients with suicidal ideation were permanently adherent to protective measures, while this frequency was significantly lower among patients without suicidal ideation and attempt. These results suggested the importance of COVID-19-related behavior, including the presence of fear of being infected, which study subjects reported, on suicidal behavior (suicide ideation and attempts).

The results related to the marital and offspring status in the group of patients with suicidal attempts are not in agreement with the results of Dehara et al. [35], which showed the association between parenthood and lower suicide risk. As we mentioned earlier, employed subjects, married, and those with children might be under more distress due to family responsibility and possible economic repercussions of COVID-19 pandemic worldwide. The presence of fear of losing employment and financial stressors are well-recognized risk factors for suicide [36].

The frequency of the diagnosis of adjustment disorder was higher among patients with suicide attempts compared with patients with suicide ideation and especially among patients without suicidal ideation and attempt, and the difference was significant. As mentioned earlier, these results indicate the importance of psychosocial implications of COVID-19 on subjects, who have never had psychiatric illness and treatment. Acute transitive psychotic disorder and delusional disorders were significantly less frequent (*p* < 0.001) in patients with suicide ideation and the patients with suicide attempts compared to the patients without suicidal ideation and attempt. Recurrent depression disorder in patients with suicide ideation and patients with suicide attempts was significantly higher (*p* < 0.001) compared to patients without suicidal ideation and attempt. It is well established that psychiatric disorders, especially with regard to mood disorders, are major contributors to suicidal behaviors [37]. Personality disorder in patients with suicidal ideation was significantly higher (*p* < 0.001), compared to patients without suicidal ideation and attempt. Ansell et al. [38] highlighted the unique effects of the severity personality disorder predicting suicide attempts over 10 years. The rest of sociodemographic and clinical characteristics, presented in Table 2, showed no significant differences between the evaluated groups. Finally, we tried to compare all patients with suicide attempts, regarding the age of admission. The frequency of psychiatric untreated patients was significantly higher among patients with suicide attempt during 2020 compared to patients with suicide attempt during 2019. The frequency of the diagnosis of adjustment disorder was more frequent among patients with suicide attempts during 2020 compared to patients with suicide attempts during 2019 and especially during 2018, when there were no patients with the mentioned diagnosis, and this difference was found to be significant. These results also indicate the psychosocial importance of COVID-19 on subjects, who have never had psychiatry illness and treatment before hospital admission and developed adjustment disorder due to COVID-19-related stress [39].

## 6 Limitations

The present study has several limitations. The retrospective design of the study could have led to a certain degree of bias in the collection of some variables. We used the cross-sectional design, and conducting a longitudinal study would be necessary to determine a causal link and long-term effects of the COVID-19 pandemic on suicidal behavior. The small sample size refers to a single psychiatric clinic and may not represent other psychiatric hospitals in Serbia. Moreover, larger multicenter studies must be organized to verify whether these trends may be confirmed in other realities. The psychopathology symptoms severity that led to hospitalization were not quantified using psychometric tools, and the presence of any other psychopathology dimensions that could have affected suicide ideation and suicide attempts should be studied. It was not possible to confirm a link, only the association of COVID-19 pandemic and suicidality, as well as reactions to restrictions (fear of contagion, isolation, and economic problems). Furthermore, the lethality of the suicide attempts during the study period in 2020 was not included in this study.

## 7 Conclusion

COVID-19 pandemic is a social stress factor that can trigger serious mental disorders among previously mental health individuals, as well as among subjects with previous psychiatric disorders, which might experience worsening symptoms and developing new mental health problems, all associated with the increased suicide risk. Our results demonstrate that were psychiatric admissions at Psychiatric Clinic Nis, Serbia, subjects with suicidal ideation were markedly more frequent, young, employed, patients with children, as well as subjects who have never had psychiatry illness and treatment before hospital admission (including psychopharamacy treatment) were more distressed due to psychosocial and economic repercussion of COVID-19 pandemic. Our results suggested the importance of COVID-19-related behavior, including the presence of fear of being contaminated and adherence to protective measures that study subjects reported, on suicidal behavior (suicide ideation and attempts).

Since studies on previous pandemics have suggested that an increase in psychiatric disorders and suicidal ideations, as a strong predictor of subsequent attempts, as well as suicide attempts may appear months after the epidemic, clinicians should investigate and monitor suicide risk in patients with psychiatric disorders over long periods of time, and it is necessary and important to protect these individuals who are a vulnerable group and provide the health services they need. Suicide prevention in the COVID-19 era is an important and difficult issue. Further research studies are needed to know how mental health consequences can be mitigated during and after the COVID-19 pandemic. Also, it would be interesting to compare the data from similar further studies from Balkan/Mediterranean region because of cultural similarities.
